# New Wistar Kyoto and spontaneously hypertensive rat transgenic models with ubiquitous expression of green fluorescent protein

**DOI:** 10.1242/dmm.024208

**Published:** 2016-04-01

**Authors:** Ana Isabel Garcia Diaz, Ben Moyon, Philip M. Coan, Neza Alfazema, Lara Venda, Kevin Woollard, Tim Aitman

**Affiliations:** 1 Division of Immunology and Inflammation, Imperial College London, London W2 1PG, UK; 2 MRC Clinical Sciences Centre andDepartment of Medicine, Imperial College London, London W12 0NN, UK; 3 Embryonic Stem Cell and Transgenics Facility, MRC Clinical Sciences Centre, Imperial College London, London W12 0NN, UK; 4 Institute of Genetics and Molecular Medicine, University of Edinburgh, Edinburgh EH4 2XU, UK

**Keywords:** GFP, SHR, WKY, Rat, Transgenics

## Abstract

The Wistar Kyoto (WKY) rat and the spontaneously hypertensive (SHR) rat inbred strains are well-established models for human crescentic glomerulonephritis (CRGN) and metabolic syndrome, respectively. Novel transgenic (Tg) strains add research opportunities and increase scientific value to well-established rat models. We have created two novel Tg strains using Sleeping Beauty transposon germline transgenesis, ubiquitously expressing green fluorescent protein (GFP) under the rat elongation factor 1 alpha (*EF1a*) promoter on the WKY and SHR genetic backgrounds. The Sleeping Beauty system functioned with high transgenesis efficiency; 75% of new rats born after embryo microinjections were transgene positive. By ligation-mediated PCR, we located the genome integration sites, confirming no exonic disruption and defining a single or low copy number of the transgenes in the new WKY-GFP and SHR-GFP Tg lines. We report GFP-bright expression in embryos, tissues and organs in both lines and show preliminary *in vitro* and *in vivo* imaging data that demonstrate the utility of the new GFP-expressing lines for adoptive transfer, transplantation and fate mapping studies of CRGN, metabolic syndrome and other traits for which these strains have been extensively studied over the past four decades.

## Abstract (precis)

**Summary:** We have created two transgenic models expressing ubiquitous GFP in WKY and SHR rat lines to support *in vivo* and *ex vivo* studies. We show their characterisation and discuss their potential use for translational research.

## INTRODUCTION

The rat has long been an important physiological model of complex human disease (Aitman et al., 2008; Iannaccone and Jacob, 2009). Two well-studied strains, both originating from Wistar stock, are the Wistar Kyoto (WKY) and spontaneously hypertensive (SHR) inbred rats (Kurtz and Morris, 1987; Okamoto and Aoki, 1963). The WKY rat has been studied extensively as a model of crescentic glomerulonephritis (Aitman et al., 2006; Atanur et al., 2013; Behmoaras et al., 2008, 2015; D'Souza et al., 2013; Kanno et al., 1998; Sado et al., 1984; Smith et al., 2007), as a control for the SHR strain and as a model of depression and behavioural abnormalities (Will et al., 2003). The SHR strain has been widely used as a model of the human hypertension and metabolic syndrome as well as a large number of other pathophysiological phenotypes including cardiac hypertrophy and failure, insulin resistance and defects in lipid metabolism (Aitman et al., 1999, 1997; Lepretre et al., 2004; Pravenec et al., 2008; Yamori and Okamoto, 1974).

Both inbred strains are well characterised, with 199 and 197 quantitative trait loci (QTLs) mapped, respectively, in WKY and SHR (Shimoyama et al., 2015), and whole genome sequences of both strains now publically available (Aitman et al., 2008; Atanur et al., 2010, 2013; Hubner et al., 2005; Shimoyama et al., 2015). With the advent of gene targeting technologies that are suited to use in rats, including zinc finger nucleases (ZFNs) (Geurts et al., 2009), transcription activator-like effector nucleases (TALENs) (Tesson et al., 2011), clustered regularly interspaced short palindromic repeats (CRISPRs) (Shen et al., 2013) and Sleeping Beauty (SB) transposon system (Ivics et al., 2009; Kitada et al., 2007; Mátés, 2011), increasing numbers of new transgenic (Tg), knockout and knock-in rat models have been created to elucidate the functional basis of disease phenotypes (Jacob et al., 2010).

Fluorescence imaging for *in vitro* and *in vivo* analysis of biological processes, combined with GFP Tg models, are valuable reagents for translational research. Tg models reporting ubiquitous enhanced green fluorescent protein (GFP) have been described in inbred rats mostly under the control of the chimeric CAG (cytomegalovirus enhancer, chicken β-actin enhancer-promoter, and intronic sequences from rabbit β-globin) promoter, using classical transgenesis (Hakamata et al., 2001), lentivirus integrations (Michalkiewicz et al., 2007) and Sleeping Beauty (Katter et al., 2013). However, there are shortcomings in some of these approaches. Classical transgenesis (Charreau et al., 1996; Mullins et al., 1990) has low efficiency and is likely to insert concatemer transgene copies in the genome. This can predispose to gene silencing and a high frequency of mosaic founders (Bishop and Smith, 1989; Garrick et al., 1998; Whitelaw et al., 1993). Lentivirus integrations have high efficiency, but also have drawbacks including triggering of transgene silencing by epigenetic regulation and production of mosaic founders. As well as limitations in transgene size, lentiviruses can cause embryo toxicity resulting from preferential transgene insertion in endogenous genes (Ellis, 2005; Hofmann et al., 2006; Lois et al., 2002; Wolf and Goff, 2009).

This paper describes the creation of two new ubiquitously expressing GFP models in WKY and SHR inbred rat lines by combining a highly efficient transgenic system and a strong mammalian endogenous promoter. We took advantage of the Sleeping Beauty (SB) transposon system that randomly integrates single copies or low copy number of a gene of interest (Ivics et al., 2014; Mátés, 2011). We opted for the rat elongation factor 1 alpha (*EF1a*) promoter (Kim et al., 1990; Mizushima and Nagata, 1990). The rat *EF1a* gene encodes an isoform of the alpha subunit of the elongation factor-1 complex, which is responsible for the enzymatic delivery of aminoacyl tRNAs to the ribosome (Sasikumar et al., 2012) and its promoter has been successfully used in gene therapy studies as a non-viral alternative to the cytomegalovirus promoter (Gill et al., 2001; Serafini et al., 2004; Zheng and Baum, 2005). We report GFP expression in embryos, tissues, *ex vivo* cell cultures and in an *in vivo* imaging model of bone marrow transplantation to validate these lines as useful tools for translational research.

## RESULTS

### Microinjection results

Seventy-five percent of newborn pups after microinjections were GFP-positive by direct inspection under UV light and by PCR. PCR assay results corresponded exactly to GFP expression by direct inspection in both WKY-GFP and SHR-GFP rat lines (Table 1).

**Table 1.**
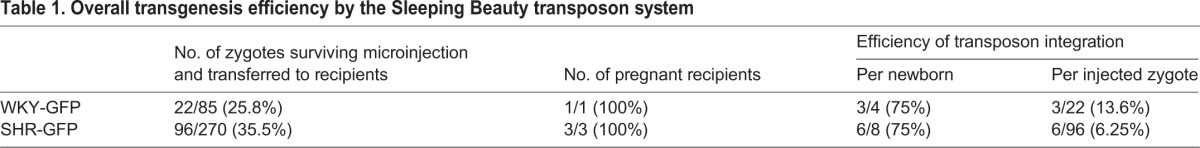
**Overall transgenesis efficiency by the Sleeping Beauty transposon system**

Two positive F_0_ founders from each strain were crossed to wild type to confirm transgene germ line transmission. All F_0_ founders transmitted the transgene. Only one high-GFP-expresser F_0_ per strain was used to derive each transgenic (Tg) rat line. Both lines showed normal growth, were able to reproduce and germline transmit the transgene, and after more than five generations, GFP expression was maintained without any sign of transgene silencing.

### Examination of the GFP integration site

Ligation-mediated PCR (LMPCR) protocol (Ivics et al., 2014) was used to locate the transgene integration site in the host genome. GFP transgene insertion sites were located using Ensembl genome browser, Rat (Rnor_6.0).

**Fig. 1.**
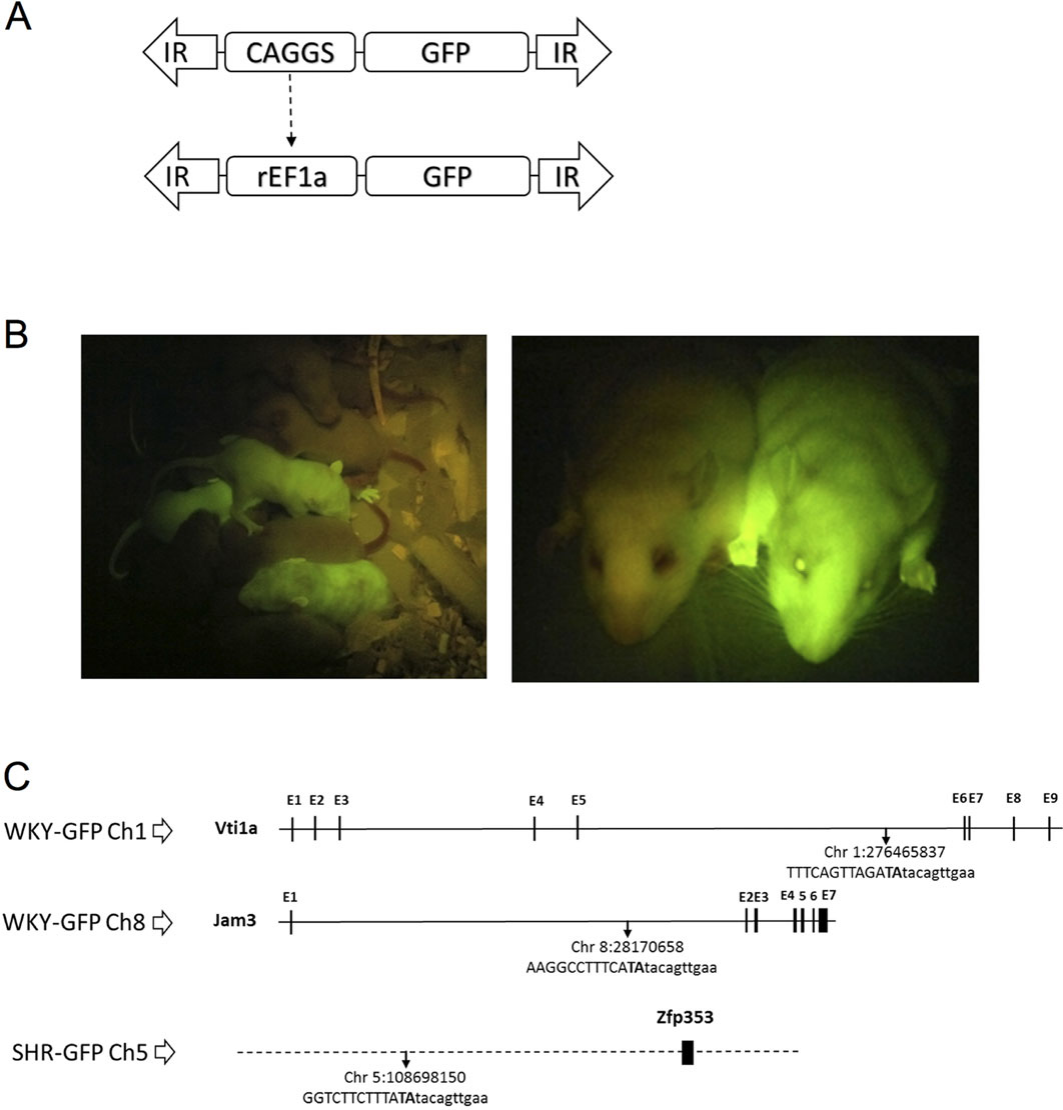
**GFP transgenic rat design.** (A) Schematic plasmid representation. Rat elongation factor 1 alpha promoter (rEF1a) replaces CAG promoter (CAGGS). IR, inverted repeats; GFP, green fluorescent protein cDNA. (B) Photograph of WKY-GFP pups (left) and adult (right) rats under excitation light 489 nm, showing wild-type and GFP Tg animals. (C) Schematic genome locus showing TA integration sites location of transgene for SHR-GFP in chromosome 5, and WKY-GFP in chromosome 1 and 8. Genomic sequence (left junction) in capitals and transgene in lowercase, dotted horizontal line refers to intergenic, continuous black line to intronic, and vertical blocks to exonic sequences.

Two integration sites were identified in the WKY-GFP founder of the Tg line on chromosome 8:28170658 and chromosome 1:276465837, both located in intronic gene areas, *Jam3* (ENSRNOG00000009149) intron 1 and *Vti1a* (ENSRNOG00000042786) intron 6, respectively (Fig. 1C). The *Jam3* intron 1 is 51,319 bp long, and the transgene resides at 37.8 kb from exon 1 and 13.5 kb from exon 2. In *Vti1a* intron 6, 102,565 bp long, the transgene is located at 81.4 kb from exon 5 and 21.1 kb from exon 6.

One single location was identified in the SHR-GFP founder of the Tg line on chromosome 5:108698150, an intergenic area where the closest gene, *Zfp353* (ENSRNOG00000050804), is located at 47.9 kb downstream (Fig. 1C).

### GFP expression in embryos, tissues and blood

**Fig. 2.**
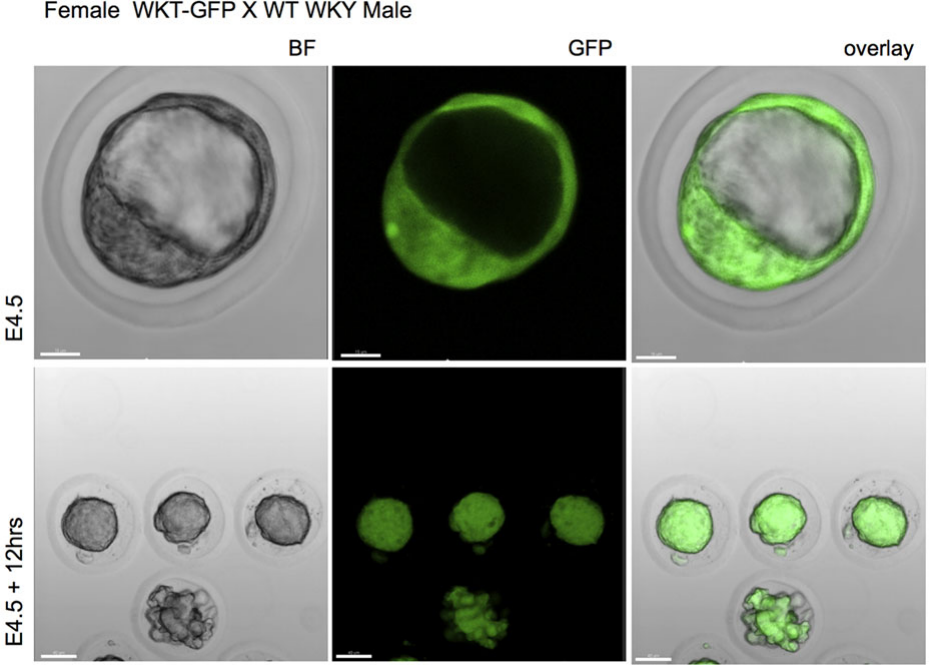
**GFP expression in embryos.** Female WKT-GFP rats were crossed with wild-type WKY male rats and one-cell embryos removed and imaged under confocal microscope. Representation of three experiments from one-cell embryos at E4.5 and E4.5 plus 12 h showing bright field (BF) images and expression of GFP. Scale bar: 15 μm (top panel); 40 μm (bottom panel).

To examine the level of GFP expression in tissues from Tg WKY and SHR rats, blood, cells and organs were processed for fluorescence microscopy. Fig. 2 shows representative examples of GFP expression in embryonic day (E)4.5 early blastocyst embryos from homozygote WKY-GFP female crossed with wild-type WKY male. All embryos were GFP-bright that sustained for at least 12 h in culture (Fig. 2, lower panel). Similar expression was noted in WKY-GFP homozygotes and SHR-GFP rats (data not shown).

**Fig. 3.**
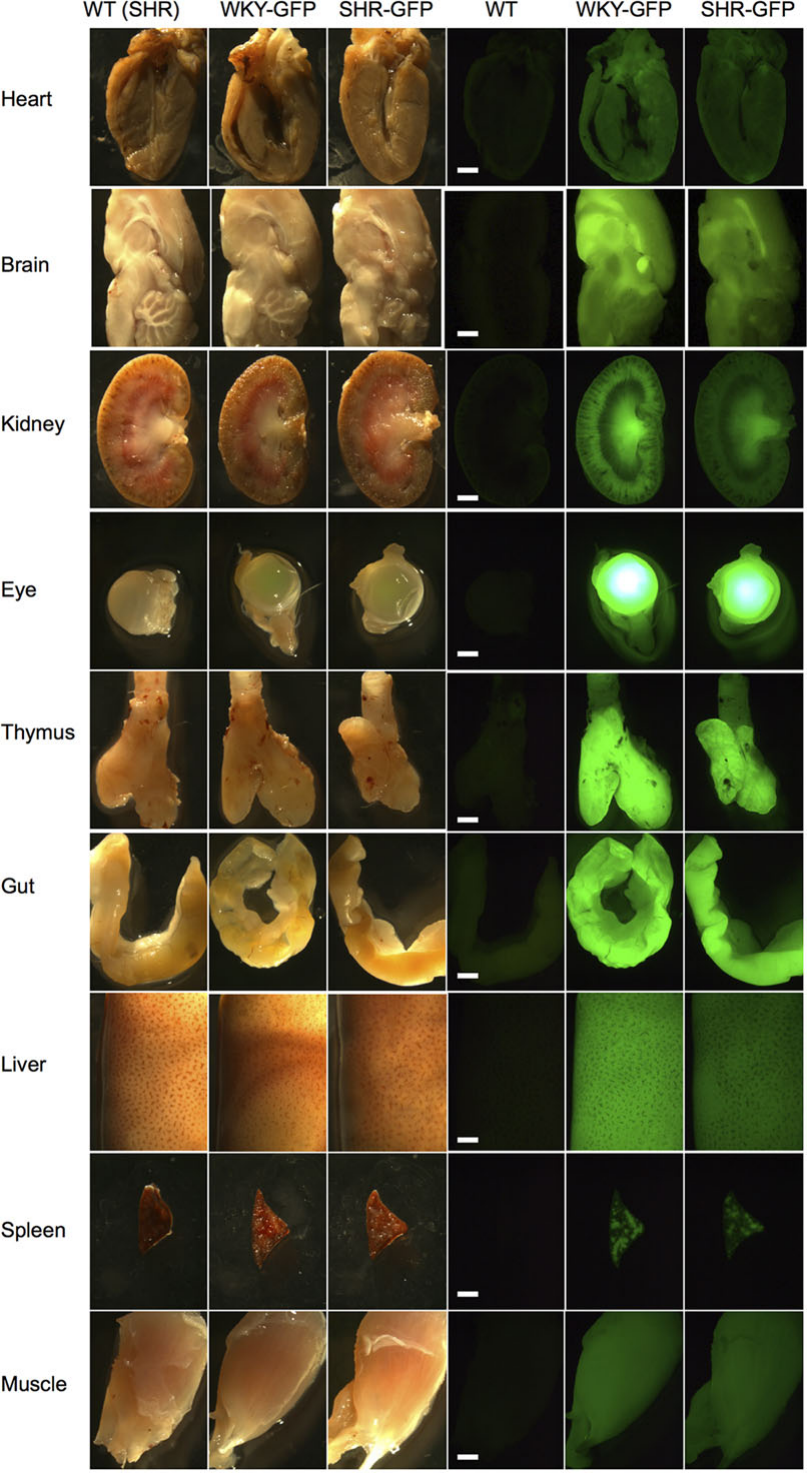
**GFP expression in organs and tissues.** Wild type (from SHR strain), WKY-GFP and SHR-GFP rats were examined for gross GFP expression in dissected heart, brain, kidney, eyes, thymus, gut, liver, spleen and muscle tissues. Whole organ extracts were mounted for white light (left three panels) and stereo-fluorescence imaging (right three panels). Scale bar: 2 mm.

We next isolated brain, eye, salivary glands, thymus, heart, lung, liver, kidney, adrenal glands, gut, pancreas, spleen, and quadriceps from wild-type (SHR), WKY-GFP and SHR-GFP rats. All organs displayed bright GFP expression with some differences in GFP intensity between and within organs (Fig. 3; Fig. S1). In general, GFP intensity was moderately less in SHR rats, although both strains expressed GFP in all organs and tissues investigated (Fig. 3). Interestingly, small variations in GFP expression were observed in heart, kidney, thymus and brain tissue, localised to specific areas within the organs (Fig. 3). This might reflect differences in *EF1a* expression in different cell types.

GFP intensity was also maintained after tissue histological processing. Kidney, liver, spleen and thymus were processed for frozen sectioning and stained for GFP and DAPI expression. All tissues were GFP-bright, with some differences in GFP expression across the tissue (Fig. S2).

**Fig. 4.**
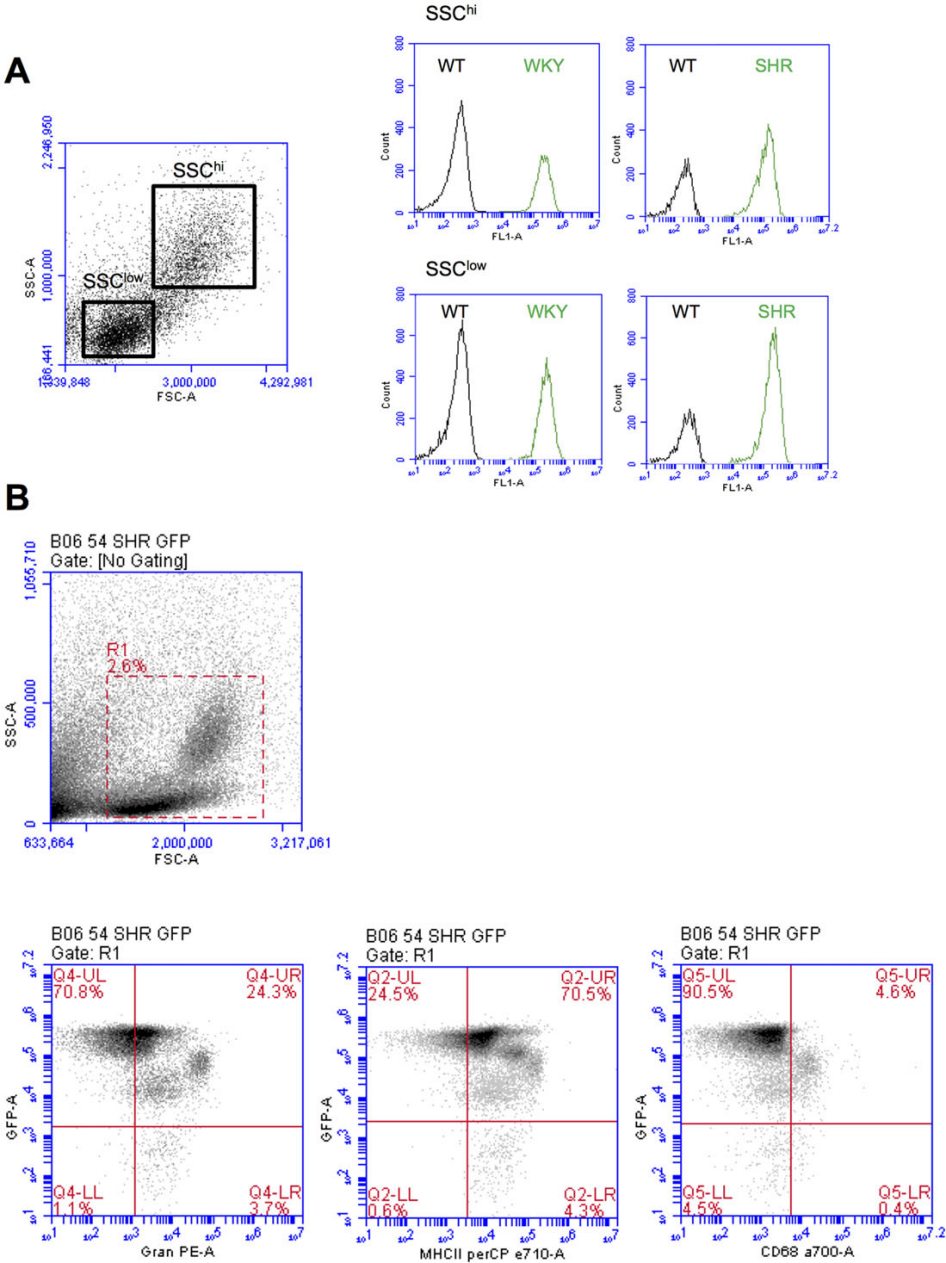
**GFP expression in blood leukocyte populations.** Anticoagulated blood from wild-type, WKY-GFP and SHR-GFP rats were examined for GFP expression with or without fluorescent-labelled antibodies using flow cytometry. (A) Histograms of GFP expression in SSC^hi^ and SSC^low^ populations. (B) Lower panels: dot plots of GFP expression versus granulocyte (Gran)-, MHC class II (MHC-II)- or CD68-positive populations, in cells gated by R1 (upper panel). Representative of at least *n*=4 rats.

We next analysed GFP expression in blood cell populations using flow cytometry. All blood leukocyte populations, based on either SSC^high^ or SSC^low^ cells, expressed similar amounts of GFP intensity in both WKY-GFP and SHR-GFP rats (Fig. 4A). However, after gating for either granulocytes (Gran), MHC class II (MHC-II) or CD68 there were noticeable differences in GFP expression on some subpopulations in SHR-GFP rats (Fig. 4B). Similar data was noted for WKY-GFP rats (data not shown). CD68^neg^Gran^pos^MHC-II^high^ cells, which are most likely lymphocyte and some granulocyte populations, expressed the highest level of GFP (Fig. 4B**)**, whereas CD68^pos^ (monocytes) are GFP intermediate, and GFP^low^-expressing cells are within Gran^pos^ and MHC-II^high^ populations (Fig. 4B). The differences might reflect *EF1a* expression in different circulating subpopulations.

### Bone marrow-derived macrophage culture *in vitro*

**Fig. 5.**
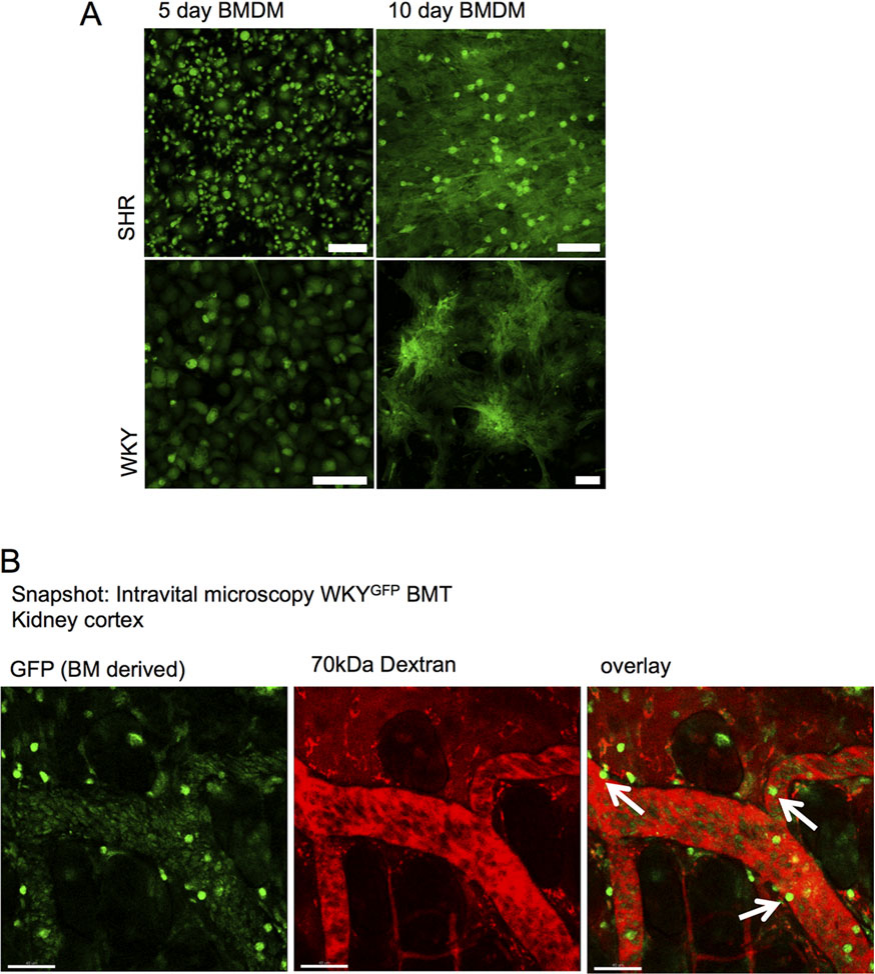
**GFP expression in BMDM and intravital microscopy.** (A) Bone marrow-derived macrophages (BMDM) were harvested and cultured for 5 or 10 days and intensity of GFP examined in WKY-GFP and SHR-GFP rats. Scale bar: 75 μm. (B). WKY rats underwent irradiation and bone marrow transplant from a WKY-GFP donor. After successful chimerisation, kidney cortex was imaged after injection of 70 kDa fluorescent dextran under anaesthesia. Snapshot is shown of GFP-positive cells interacting with endothelial surfaces within the kidney (arrows). Representative of at least *n*=4 rats. Scale bar: 40 μm.

To examine if GFP could be sustained in primary cell culture passage, which would be useful for downstream experimental applications, we examined GFP expression in bone marrow-derived macrophages (BMDM). BMDMs cultured over 5 and 10 days sustained high GFP expression, as detected by fluorescence microscopy (Fig. 5A). There was no difference in WKY-GFP or SHR-GFP BMDM GFP expression, and similar amounts of GFP were expressed between 5 and 10 days (Fig. 5A). This indicates that within proliferating macrophage cultures, EF1a activity and consequently GFP intensity is maintained in daughter cells.

### *In vivo* bone marrow chimeras and intravital microscopy

To further examine the utility of Tg rat GFP lines in potential disease models and *in vivo* applications, we examined GFP intensity in WKY-GFP bone marrow chimera experiments using intravital microscopy. WKY-GFP bone marrow transfer was performed in WKY recipients and the kidney cortex exposed for confocal imaging. Fig. 5B shows that GFP-bright cells can be seen located at the endothelial interface.

## DISCUSSION

The potential of rat models for understanding basic biology and human health and disease (Aitman et al., 2008) has increased owing to availability of new gene targeting tools applicable to the rat (Atanur et al., 2013; Jacob et al., 2010) and communal efforts to increase and refine rat genomic resources. Important aims of creating Tg rat models, or any Tg animal model, are to achieve high transgenesis efficiency with strong and permanent expression of the gene of interest through generations, with high efficiency to minimise use of resources and animal numbers. The efficiency of transgenesis has increased in recent years as a result of development and refinement of new systems, from low efficiency of classical transgenesis by microinjection of naked DNA (Charreau et al., 1996; Filipiak and Saunders, 2006) to higher efficiency with retroviral transgenesis and the latest lentivirus vectors (Michalkiewicz et al., 2007). The Sleeping Beauty transposon system has advantages for germline transgenesis as a simpler and safer delivery system, matching or exceeding other approaches. This approach, with a single copy of a permanent insertion in the genome, leads to preferential intergenic integrations and is less prone to silencing (Ivics et al., 2014; Katter et al., 2013; Mátés, 2011; Mátés et al., 2009; Park, 2007).

We have created two new transgenic lines, on the WKY and SHR genetic backgrounds, that display and take advantage of many of these characteristics. In our experiments, 75% of F_0_ newborn pups were transgene-positive (Table 1), one of the highest transgenesis efficiencies reported. Such efficiency, in our case, seems independent of the SHR or WKY genetic rat background. In our Tg models, GFP expression seems to be independent of integration site and genomic background and dependent on promoter endogenous/native activity. Further, expression was transmitted unchanged through more than five generations of breeding and through 5-10 days of *in vitro* macrophage culture, demonstrating the stability of expression from the transgene constructs integrated into the germline genome. Finally, the high level of GFP expression was shown to have created a suitable platform for cell fate tracking through intravital microscopy and confocal imaging.

Avoiding the production of mosaic founders is an important way to minimise cost and increase functional efficiency. Sleeping Beauty is a plasmid/mRNA-based method where the transposase mRNA does not need embryonic genome activation. In rats this begins at the four-cell stage and only needs translation of the transposase mRNA in the early embryo stages. This system largely avoids the creation of mosaic founders (Mátés, 2011). This is superior to lentivirus transgenesis where reverse transcription is necessary and where viral infection and integration can continue with embryonic development (Park, 2007). The transmission of the transgene over five generations in our lines excludes the possibility of mosaicism in the selected founders of our established WKY and SHR lines.

The Sleeping Beauty transposon system randomly integrates the gene of interest in the genome with a bias to intergenic integration (Ammar et al., 2012; Grabundzija et al., 2010; Katter et al., 2013; Moldt et al., 2011). We show in our new GFP Tg rat models, by locating and verifying the integration locus sites, that there was no functional gene disruption resulting from exon insertion. We confirmed individual transgene integration by locus-specific PCR, making possible the isolation of each integration by selective breeding and establishing unique locus integration per rat line. In the SHR inbred background the insertion position was at chromosome 5:108698150, an intergenic locus 47.9 kb upstream of gene *Zfp353*. In such a location, using the rat genome information available (Rnor_6.0), we cannot foresee any biological impairment. In the WKY inbred background the two located integration sites were intronic, one on chromosome 1:276465837, intron 6 (102,565 bp long) of gene *Vti1a* (vesicle transport through interaction with t-SNAREs 1A) and the other on chromosome 8:28170658, intron 1 (51,319 bp) of gene *Jam3* (junctional adhesion molecule 3). Regulatory splicing elements can be found in the intron up to 300 bp from the exon-intron border (Barash et al., 2010). Our intronic integrations are far away from these areas, being ∼13.5 kb from the closest exon-intron border. Therefore, effects on the splicing machinery would not be foreseen although interference resulting from Tg insertion in introns, which regulate gene expression by noncoding RNA molecules, intron-mediated enhancement or by other intronic regulatory functions, cannot be completely excluded.

To ensure strong and permanent expression, many factors are necessary, including a strong promoter and fortuitous integration in the genome to avoid transgene silencing (Kioussis and Festenstein, 1997). The selection of promoter was fundamental to accomplish this goal. We decided to move away from the most-used, the chimeric exogenous CAG promoter, because it contains the cytomegalovirus enhancer sequence and because of its viral nature could trigger silencing mechanisms affecting GFP expression. Moreover, although CAG promoters are ubiquitous, it has been reported there are different expression patterns within different tissues and within different transgenic models, from high expression to low or no expression (Hakamata et al., 2001; Inoue et al., 2005; Katter et al., 2013; Michalkiewicz et al., 2007; Murakami and Kobayashi, 2012; Popova et al., 2008; Takeuchi et al., 2003). We therefore opted for an endogenous promoter to maximise the likelihood of permanent expression and escape from gene silencing. Most promoters that have been used have some disadvantages. For example, rat transgenic models using ubiquitin C (van den Brandt et al., 2004), phosphoglycerate kinase 1 (*PGK1*) (Remy et al., 2010) and *Rosa26* (Montanari et al., 2014) promoters report chimeric expression. Potential promoter candidates were compared using the online BioGPS application (http://biogps.org) where the tissue-specific pattern of mRNA expression of their endogenous driven protein is quantified. We hypothesized that the expression of GFP will mimic, at least to an extent, the reported expression of our gene of interest. Eukaryotic elongation factor 1 alpha 1 (EEF1A1), also known as elongation factor 1 alpha (EF1a) has one of the most wide-ranging tissue expression patterns, with the highest median (M=10,426.3), followed by ubiquitin C (M=4620.6) and PGK1 (M=4.5). One possible weakness of EF1a is the low expression in heart and skeletal muscle tissues. However, given the near ubiquitous expression, we selected the *EF1a* promoter for our Tg models.

In our Tg models, GFP expression seems to be independent of integration site and genomic background and dependent on promoter endogenous/native activity. We visually confirmed that GFP expression follows the pattern of *EF1a* mRNA expression, with a markedly lower expression in the heart, in both SHR and WKY rats, implying possibly that the *EF1a* promoter is the main determinant in these strains of the level of tissue GFP expression. It would be interesting to perform further studies to quantify *gfp* mRNA versus *EF1a* mRNA in tissues to assess transcriptional correlation. If true, these models could be used as an *EF1a* expression reporter, and also as an *EF1a* canonical function assay to deliver aa-tRNA to ribosomes in mRNA translation. It could well be a reporter for cellular translation levels. The difference in GFP intensity between the SHR and WKY Tg models could be a result of transgene copy, SHR with a single copy shows relatively lower expression of GFP than WKY with two integrations. Finally, heterogeneous GFP expression within a tissue, such as the brain, might reflect a differing chromatin state of the integration site. One hypothesis could be that, because the *EF1a* promoter is essential in cellular protein translation, integration could make the chromatin more accessible in particular cell types if these loci are normally closed when translation occurs. Further work is needed to confirm whether these models reflect *EF1a* expression in specific tissues and/or chromatin state after integration.

In summary, we have created two novel Tg models expressing ubiquitous green fluorescent protein in WKY and SHR inbred rat lines to support *in vivo* and *ex vivo* studies in cell tracking, tissue and organ transplantation for further elucidation of the complex disease traits of human crescentic glomerulonephritis (CRGN) and metabolic syndrome, respectively. Our preliminary study using real-time imaging of kidney leukocyte-endothelial interactions in WKY-GFP bone marrow chimeras confirms the utility of GFP transgenic rats for fluorescent imaging. Both novel Tg lines have considerable value for future translational research in the scientific community.

## MATERIALS AND METHODS

All animals were housed in individually ventilated cages. All procedures were carried out according to the institutional guidelines for the care and use of experimental animals and the ARRIVE guidelines. Animal studies were approved by the UK Home Office.

### Generation of ubiquitous GFP WKY and SHR rats using Sleeping Beauty transgenesis

#### Preparation of the transposon donor plasmid and microinjection

Schematic of plasmid is shown in Fig. 1A. The CAGGS promoter in pSB IR-DR(L)-CAGGS-eGFP-pA-IR-DR(R) plasmid was replaced with the native rat elongation factor-1 alpha promoter (*EF1a*) from pDRIVE-rEF1α (InvivoGen). Fragment *Pst*I-*Nco*I containing the *EF1a* promoter was blunted and inserted into the *Not*I-*Mlu*I blunted backbone. Screening for ‘sense’ orientation was by *Eco*RI digestion. Functional elements of the Sleeping Beauty construct were confirmed by sequencing. The SB100x transposase was prepared following protocol by Mátés (2011).

#### Synchronization of oestrus cycle in rat recipients

The foster females aged 8 to 12 weeks were injected intraperitoneally (i.p.) with 40 μg of luteinizing hormone-releasing hormone (LH-RH) agonist 4 days prior to mating. On the day of mating, the recipients were individually placed with vasectomised males. The following day, the females were de-mated and examined for the presence of copulation plug. The females presenting a plug were used as embryo recipient for embryo transfer surgery on the same day.

#### Embryo preparation and microinjection

Six SHR and nine WKY females aged 4 to 6 weeks old were used as embryo donors. Two days prior to mating, females were injected i.p. with 30 IU of pregnant mare serum gonadotropin (PMSG). On the day of mating, the females were injected i.p. with 40 IU of human pregnancy urine chorionic gonadotropin (HCG) and placed individually with SHR or WKY males. The following day, the females were separated, culled and the oviducts removed. The oviducts were then placed in M2 media (Sigma) and the ampulla torn to release single-cell embryos. Hyaluronidase was then added to the media to remove the cumulus cells. The embryos were collected and cleaned using a mouth pipette. The fertilized embryos were transferred from M2 to KSOMaa media (Zenith Biotech) and left in the incubator at 37°C, 5% CO_2_ until they were used for pronuclear injections and embryo transfer (20-40 embryos per female) using standard protocols (Filipiak and Saunders, 2006; Geurts et al., 2009). Briefly, pronuclear injection of mixed donor plasmid (0.4 ng/μl) and mRNA SB100X transposase (5 ng/μl) was performed in WKY and SHR rat one-cell embryos. Injected embryos were transferred to the oviduct of day-0.5 pseudopregnant rats and then replaced into Sprague Dawley rat recipients by embryo transfer surgery.

### Screening of GFP-positive pups

Selected F_0_ founders were crossed with wild types; F_1_ were screened by PCR and GFP-positive animals were further screened for GFP expression in blood to choose high expressers for breeding. Genomic DNA was extracted from ear clips by overnight proteinase K digestion at 56°C and precipitated next day with isopropanol. PCR with two primer pairs was performed to detect 343 bp actin fragment as control and 180 bp GFP fragment as transgene positive: DNA_rat_bActin_F: 5′-TGTGTTGTCCCTGTATGCCTCT-3′; DNA_rat_bActin_R: 5′-ATTGCCGATAGTGATGACCTGA-3′; GFP_F: 5′-GGCACCTACCCCAGCGGCTA-3′; GFP_R: 5′-CCGGTGCCCACCACCTTGAA-3′.

PCR cycle conditions were: 95°C for 2 min, 30 cycles of 95°C for 30 s, 60°C for 30 s, and 72°C for 30 s with a final 5-min elongation at 72°C. Tail blood was collected into lithium-heparin microvette tubes and GFP blood expression was assessed using an AccuriC6 (BD Biosciences) flow cytometer (see ‘Detection of GFP expression’ below). Whole-animal GFP expression was confirmed under an excitation light (489 nm) (Fig. 1B).

### Integration site identification and locus confirmation

Ligation-mediated PCR (LMPCR) (Ivics et al., 2014) was used to locate the transgene integration site in the host genome. We designed primers to combine with Tbal 5′-CTTGTGTCATGCACAAAGTAGATGTCC-3′ from the LMPCR protocol to verify individual transgene integration by locus-specific PCR: Ch1_is_F: 5′-GACACATCCCTTGCTGTGGA-3′; Ch1_is_R: 5′-GCTATAACAAAGGGGACAGGCT-3′; Ch5_is_F: 5′-CTCCAGCTTGCTTCTTGGGA-3′; Ch5_is_R: 5′-ACTGCACCCCAGAAAAAGCA-3′; Ch8_is_F: 5′-GAAACACTGCACGTGGTGAC-3′; Ch8_is_R: 5′-TCTCTTCCCACAGCCTTTCC-3′.

### Detection of GFP expression

#### Embryos

E4.5 embryos were collected and carefully placed in glass-bottom petri dishes for confocal imaging, using a Leica Sp5 microscope. Some embryos at E4.5 were harvested and cultured overnight (12 h) in M2 media before imaging.

#### Organs

Brain, eye, salivary gland, thymus, heart, lung, liver, kidney, adrenal gland, gut, pancreas, spleen and quadraceps were dissected, washed, fixed (4% PFA 30 min) and mounted on open glass slides for imaging using a Leica MZ16F fluorescence stereoscope and Leica camera DFC420C. For some organs, longitudinal dissection was used to show GFP intensity of inner organ structures. In addition, kidney, liver, spleen and thymus gross dissections were embedded in OCT and 2-μm frozen sections mounted onto slides using hard set mounting media with DAPI (Vectashield). Sections were imaged using an upright fluorescent microscope (Olympus) for GFP and DAPI.

#### Blood

Blood was collected into lithium-heparin microvette tubes from tail bleeds. Red blood cells were lysed (155 mM NH_4_Cl, 12 mM NaHCO_3_, 100 mM EDTA) and leukocytes washed in PBS before permeabilisation (fix-Perm kit, Invitrogen) and incubation with antibodies against rat Gran-PE (HIS48), MHC-II-perCP (HIS19) and CD68-Alexa Fluor 700 (ED1) (all 1:50; BioLegend). Cells were analysed by flow cytometry using an AccuriC6 (Becton Dickinson) with appropriate colour compensation and gates set to unstained cells.

### Culture of bone marrow-derived macrophages

Rat bone marrow-derived macrophages were isolated from femurs and differentiated using RPMI 1640 supplemented with 10% fetal bovine serum (Hyclone) and 5 ng/ml Rat M-CSF (Peprotech) as previously described (D'Souza et al., 2013). Cells were cultured in 12-well plates for 5 or 10 days at 37°C, 5% CO_2_ and GFP fluorescence images were taken using confocal microscopy.

### Intravital microscopy

To examine utility of GFP Tg rats for translational work, we tested the real-time imaging of kidney leukocyte-endothelial interactions using intravital microscopy in WKY-GFP bone marrow chimeras. WKY-GFP bone marrow was harvested and injected into WKY wild-type recipients to create bone marrow chimeras as previously described (Smith et al., 2007). Briefly, femurs were harvested from WKY-GFP rats and bone marrow cells collected under sterile conditions. Recipient WKY rats were irradiated (8 Gy) and bone marrow cells (∼8×10^6^) injected intravenously (i.v.). After 8 weeks, reconstitution was confirmed (data not shown) and kidneys imaged under anaesthesia, after exposure of superficial kidney cortex from surrounding tissue by careful blunt dissection. Images were taken using SP5 confocal microscope with i.v. infusion of 70 kDa dextran-tetramethylrhodamine (Invitrogen).
